# Navigating the data-scarce landscape of pediatric hyperkalemia: a perspective on sodium zirconium cyclosilicate

**DOI:** 10.1007/s00467-026-07333-3

**Published:** 2026-04-30

**Authors:** Lucas Maciel de Almeida Corrêa, Clara Belo Gamon Santiago, Alexandre de Assis Barbosa, Luiggi Kevin Virgino Brandão, Camilla Cristina Silva Fernandes, Letícia Mantovani Milan, Paula Ramos Carneiro

**Affiliations:** 1 Faculdade de Medicina de São José do Rio Preto (FAMERP), Avenida Brigadeiro Faria Lima, 5544, 15090-000 São José do Rio Preto, SP Brazil; 2 Universidade Estadual de Mato Grosso do Sul (UEMS), Campo Grande, Brazil; 3 Centro Universitário Uninorte (UNINORTE), Rio Branco, Brazil; 4 Universidade do Estado da Bahia (UNEB), Salvador, Brazil

**Keywords:** Hyperkalemia, Sodium zirconium cyclosilicate, Pediatrics, Chronic kidney disease, Renin–angiotensin system inhibitors

## Abstract

For decades, pediatric hyperkalemia has been managed largely with non-selective cation-exchange resins, despite limitations in tolerability, palatability, and gastrointestinal safety. This perspective reviews the role of sodium zirconium cyclosilicate (SZC) in children and places the pediatric evidence in the context of broader hyperkalemia management. Current pediatric SZC data remain sparse and are derived from a small retrospective cohort of children with chronic kidney disease (CKD) stages 4–5/5D and an in vitro infant formula pretreatment study. Within those constraints, SZC was associated with potassium reduction in acute and chronic settings, favorable tolerability, stable serum sodium and blood pressure, and high acceptability, which is relevant in pediatrics, where palatability and adherence often determine effectiveness. The available literature also suggests practical pediatric applications, including age-based oral dosing and formula-pretreatment strategies, although no validated weight-based regimen has been established. Beyond potassium lowering, SZC may support a more individualized approach to potassium management in selected children, potentially reducing the need for broad dietary restriction and helping preserve renin–angiotensin–aldosterone system inhibitor therapy when hyperkalemia becomes a limiting factor. However, these broader clinical implications remain preliminary, largely observational, and partly extrapolated from adult data. Accordingly, SZC should not yet be viewed as an established pediatric practice standard, but rather as a promising adjunct for carefully selected patients in whom older resins are poorly tolerated or ongoing hyperkalemia compromises diet quality or cardiorenal therapy. Until prospective pediatric trials better define dosing, safety, and patient-centered outcomes, any off-label use should remain individualized and closely monitored.

**Graphical abstract:**

**Figure:**
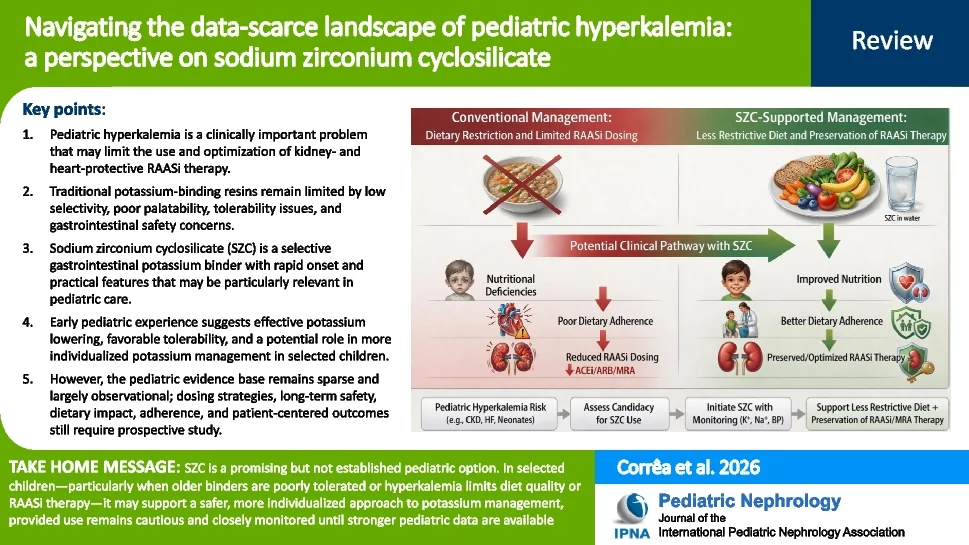
A higher resolution version of the Graphical abstract is available as Supplementary information

**Supplementary Information:**

The online version contains supplementary material available at https://doi.org/10.1007/s00467-026-07333-3.

## The clinical dilemma

The management of hyperkalemia in the pediatric population remains a clinically relevant challenge for pediatric nephrologists and cardiologists alike. Disease-modifying therapies—specifically angiotensin-converting enzyme inhibitors (ACEIs), angiotensin receptor blockers (ARBs), and mineralocorticoid receptor antagonists (MRAs)—play important roles in delaying the progression of chronic kidney disease (CKD) and improving outcomes in heart failure (HF) [1]. Yet, the inherent risk of hyperkalemia associated with these agents frequently necessitates dose reduction or outright discontinuation, thereby compromising therapeutic efficacy [1]. Indeed, in children with CKD, the administration of ACEIs or ARBs amplifies the risk of hyperkalemia by more than twofold (2.14-fold increase) [2].

In the realm of pediatric cardiology, while direct data remain sparse, cohort studies in HF demonstrate that ACEI usage significantly elevates hyperkalemia risk (OR 1.70), with renal insufficiency (OR 3.81) and diabetes (OR 1.52) emerging as the dominant predictors [3]. Furthermore, hyperkalemia may limit optimal dosing, restricting target doses of ACEI/ARB in 4.6% of patients and MRAs in 15% of HF cases, and leading to MRA discontinuation in up to one-third (33.3%) of patients [1].

Children at higher risk include those with advanced CKD, particularly of glomerular etiology, in whom hyperkalemia prevalence reaches 18.8% in stages 4–5 and 14.3% during visits for glomerular disease [2]. In the neonatal intensive care setting, extremely low birth weight (ELBW) premature infants are also at risk for non-oliguric hyperkalemia (NOHK), with reported incidence ranging from 11 to 52% within the first 48 h of life [4]. Case reports further illustrate this phenotype in late preterm contexts and severe perinatal stressors [5]. From a neonatal endocrine perspective, classic congenital adrenal hyperplasia (CAH) may present with both hyponatremia and hyperkalemia [6, 7]. Rare tubulopathies, such as pseudohypoaldosteronism type 1 (PHA1), also predispose neonates to severe hyperkalemic episodes [8]. Additionally, comorbidities such as diabetes and cardiovascular diseases significantly compound the risk of hyperkalemia in the pediatric cohort [9].

## Pharmacology and mechanism: Why SZC is different

Sodium zirconium cyclosilicate (SZC), commercially known as Lokelma, is a highly selective ion exchanger within the gastrointestinal tract that captures potassium ions (K +) in exchange for sodium (Na +) and hydrogen (H +) [10, 11]. It is supplied as a powder for oral suspension and is typically mixed with water immediately before administration [10–12]. In adult practice, initial correction usually uses 10 g three times daily for up to 48 h, followed by maintenance regimens of 5–15 g/day; in pediatrics, available approaches include age-based oral dosing in children with CKD or dialysis and formula pretreatment strategies in infants, but no validated weight-based regimen has yet been established [10–14]. Its rapid onset of action (approximately 1 h) and odorless, tasteless profile make it attractive for children, in whom palatability often determines adherence [10–12, 15]. By contrast, conventional potassium binders such as calcium polystyrene sulfonate (CPS) and sodium polystyrene sulfonate (SPS) are limited by poor tolerability and important gastrointestinal risks, including colonic necrosis and perforation [16–18].

Patiromer also warrants specific consideration because it is another newer binder with better tolerability than legacy resins and better palatability than SPS [12, 15]. However, the two agents are not interchangeable. Compared with patiromer, SZC offers a faster onset and greater potassium selectivity, which may be advantageous when earlier control is needed [10–12]. Patiromer exchanges potassium for calcium rather than sodium and may therefore be attractive when sodium load or edema is a major concern, whereas SZC exchanges potassium for sodium and hydrogen and requires attention to volume status, serum sodium, and blood pressure in susceptible patients [10–13, 19]. Practical differences also matter: SZC is prepared as a powder in water and should be separated from other oral medications by 2 h, whereas patiromer generally requires a 3-h separation window [12]. Thus, both agents represent an advance over SPS and CPS, but SZC may be particularly useful when rapid potassium lowering, good palatability, and flexible pediatric administration are priorities [12, 15, 19].

Infant formula pretreatment: Liu and colleagues [14] described an in-vitro strategy in which SZC was added directly to commercial formula before administration to reduce potassium content prior to ingestion. Using Kabrita (goat milk) and Similac (cow milk), SZC was added at 0.5 g/mmol K + (high-dose) or 0.25 g/mmol K + (low-dose). Potassium concentrations fell significantly within 5 min in both formulas; in the high-dose condition, Kabrita dropped from 27.48 ± 0.36 to 14.36 ± 0.54 mmol/L (approximately 47.75% reduction) and Similac from 24.92 ± 0.44 to 11.69 ± 0.37 mmol/L (approximately 53.12% reduction). Sodium rose modestly, calcium changes were small, and no meaningful effect on pH or macronutrients was reported [14]. This was a pretreatment or decanting strategy rather than direct binder administration to the infant. Compared with older resin-based approaches, the short preparation time suggests a more feasible bedside or caregiver workflow and may expand the use of commercially available formulas when low-potassium options are limited, although clinical validation in infants is still needed [12, 14, 16].

## The paucity of pediatric data: a critical unmet need

Across an extensive search spanning PubMed, Scopus, Web of Science, Embase, and Google Scholar, the direct pediatric SZC evidence base remains very small and is limited to three reports: one single-center retrospective clinical series in children with advanced CKD or dialysis, its preliminary conference abstract, and one in-vitro formula-pretreatment proof-of-concept letter. Broader pediatric hyperkalemia literature supports the clinical relevance of potassium management in CKD and provides dietary management recommendations, but SZC-specific pediatric evidence remains limited and should be interpreted as preliminary [2, 12–14, 20, 21].

### Clinical safety and efficacy

The most informative dataset is a retrospective chart review of 20 children (6 months–18 years) with CKD4–5/5D and sustained hyperkalemia treated with age-based SZC dosing (0.5–10 g per dose), rather than a standardized mg/kg regimen, and stratified into short-term (< 30 days) versus long-term (> 30 days) use. In the acute setting, serum potassium fell from 6.7 (6.1–6.9) to 4.4 (3.7–5.2) mEq/L (*p* < 0.001). In long-term use (median approximately 5 months), potassium declined from 6.1 (5.8–6.4) to 4.8 (4.2–5.4) mEq/L (*p* < 0.001), maintaining values within an acceptable range for ongoing management. Importantly, no serious edema was observed, and serum sodium and blood pressure remained stable [13]. Hypokalemia occurred in two patients (nadir 3.2 mEq/L at 72 h), underscoring the need for early monitoring and titration rather than weakening the overall safety signal [13].

### The tolerability and palatability

 Beyond biochemistry, the study assessed acceptability using a 5-point Facial Hedonic Scale. Tolerability remained favorable (average score approximately 1–2), 9/10 long-term patients gave the most positive rating for taste, and all 10/10 reported that they would take SZC again if prescribed [13]. This is clinically relevant in pediatrics, where poor adherence often limits the real-world effectiveness of potassium binders, although these acceptability data remain preliminary and derive from a small retrospective cohort [13].

## Potential clinical implications

At present, the pediatric literature supports cautious consideration of SZC in selected scenarios, but it does not yet establish definitive practice pathways for dietary liberalization, long-term renin–angiotensin–aldosterone system inhibitor (RAASi) enablement, or outcome improvement beyond potassium control [12–14, 20–24].

**Fig. 1:**
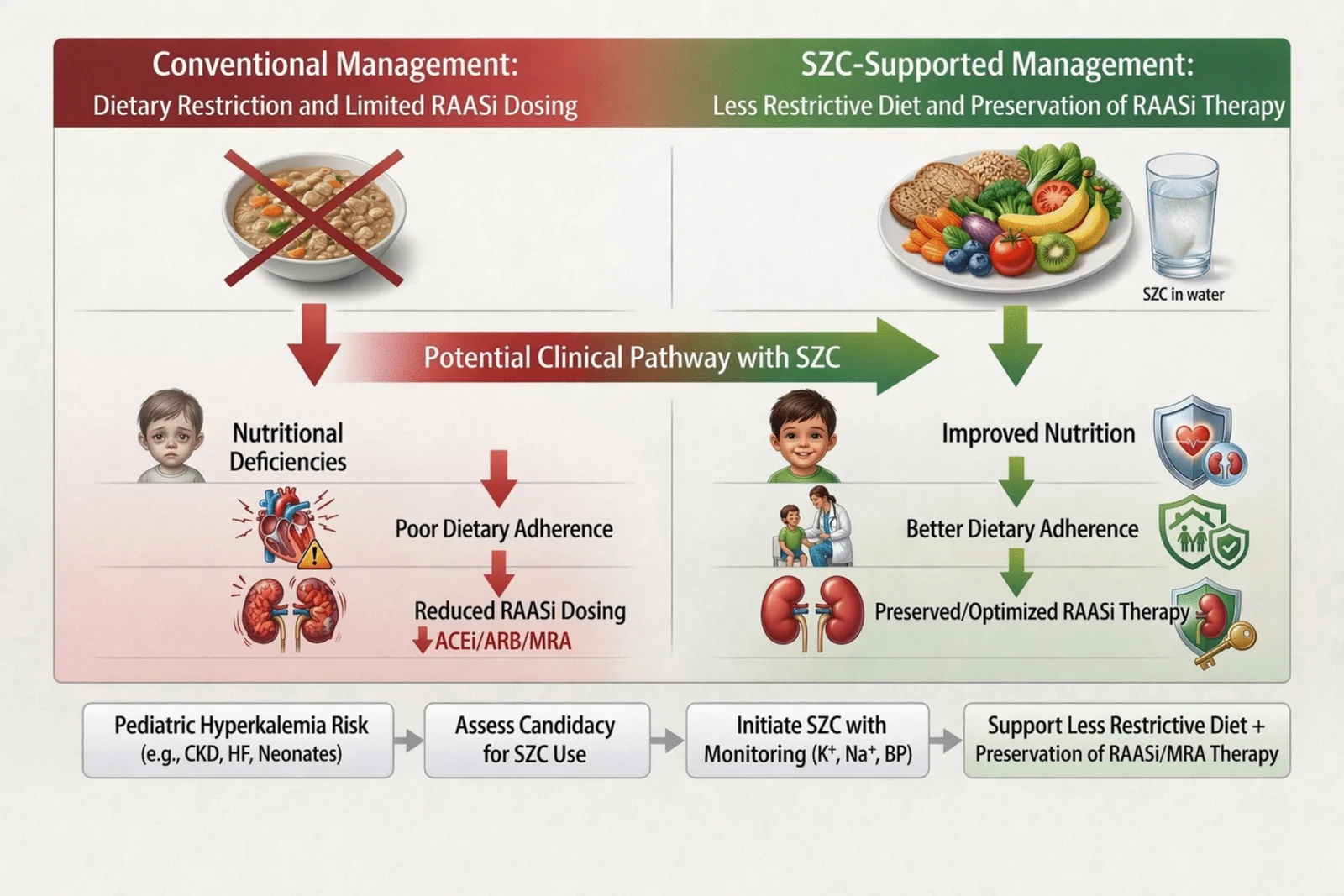
Conceptual framework for potassium management in pediatric hyperkalemia. The figure presents a conceptual model comparing a traditional restrictive strategy (left) with a more individualized SZC-based approach (right). In both arms, the pathway should be read sequentially rather than as independent parallel outcomes: dietary management influences adherence, which in turn affects preservation or reduction of RAASi therapy in selected patients

Current pediatric data suggest that SZC may help support a shift from broad chronic potassium restriction toward more individualized potassium management under close biochemical monitoring, but this should still be regarded as a preliminary and hypothesis-generating perspective rather than an established pediatric practice standard [13, 20, 21]. In the developing child, potassium restriction often results in avoidance of fruits, vegetables, and whole grains, with consequences for diet quality, family routines, socialization, and adherence [20, 21]. SZC may therefore reduce the degree of restriction required in selected patients, but pediatric evidence for dietary liberalization remains limited to early observational experience [13, 20, 21]. This was reflected in the cohort by Khandelwal et al. [13] (*n* = 20; CKD stages 4–5/5D; median age 10.8 years), in which SZC was effective and well tolerated in both acute and chronic hyperkalemia and was descriptively associated with dietary liberalization in a subset of patients, including some with prior poor adherence to restriction; however, these observations remain retrospective and exploratory [13]. These interrelated potential effects are summarized in Fig. 1.

Plant-based dietary patterns may be especially relevant in CKD because higher fiber intake may favor saccharolytic fermentation, support intestinal barrier integrity, and reduce the generation and absorption of gut-derived uremic toxins [25, 26]. Contemporary educational guidance likewise emphasizes that potassium management should be individualized and should avoid universal dietary prohibitions that degrade diet quality [25, 26]. When a pharmacological tool is available to mitigate hyperkalemic risk, the traditional conflict between plant-rich diets and potassium exposure becomes more manageable. In this framework, SZC may help reduce the tension between biochemical risk and access to a plant- and fiber-rich diet, although this remains an extrapolative and hypothesis-generating implication in pediatrics rather than a directly proven clinical outcome [13, 20, 25, 26].

Beyond nutrition, SZC may help address a recurrent therapeutic ceiling in cardiorenal populations: hyperkalemia limiting optimization of RAASi, including MRAs. In pediatrics, this is particularly relevant in two settings where suboptimal dosing may compromise target-organ protection over years: CKD with hypertension or proteinuria, and cardiac disease with ventricular dysfunction [22]. In adults, discontinuation of RAASi after hyperkalemia is associated with worse cardiorenal outcomes, supporting efforts to maintain these therapies whenever safely possible [23]. The REALIZE-K trial was designed to test whether SZC can enable rapid and sustained optimization of spironolactone in HF with elevated hyperkalemia risk, formalizing the concept that potassium control may permit continuation of high-value therapies; however, the relevance of RAASi enablement in pediatrics remains inferential and is currently extrapolated largely from adult data [22–24].

However, enthusiasm must be balanced with pharmacological and economic prudence. Because SZC exchanges K + for Na +, children with severe cardiac disease or anuria still require careful monitoring of volume status, serum sodium, and blood pressure, even though edema was not observed in the pediatric cohort [13]. SZC is also likely to have a higher acquisition cost than legacy cation-exchange resins, but adult data show that hyperkalemia itself imposes substantial healthcare costs through emergency care, hospitalization, and downstream complications [12, 27]. When SZC prevents recurrent hyperkalemia, avoids urgent dialysis or admission, and permits continued ACEI/ARB/MRA exposure, this higher upfront cost may still be clinically and economically justifiable in selected pediatric phenotypes [22, 23, 27]. The key question is not whether SZC is simply another binder, but which children are most likely to benefit, how it should be monitored in routine practice, and which outcomes should define success, including growth, diet quality, adherence, RAASi/MRA optimization, and healthcare utilization.

Indeed, Khandelwal et al. [13] highlighted the need for studies evaluating routine SZC use to improve diet and nutritional status in children with CKD. Accordingly, the next step must be pediatric, pragmatic, and mechanistic, with attention to dosing, monitoring, diet quality, adherence, and RAASi enablement. Until those data are available, SZC may be considered on an individualized off-label basis in carefully selected children when the limitations of older resins and the harms of persistent potassium restriction are weighed against the uncertainties of a pediatric evidence base that remains limited.

## Current evidence and remaining gaps

For decades, the management of pediatric hyperkalemia has relied largely on CPS or SPS, cation-exchange resins with low selectivity and limited tolerability. SZC has shown promising safety and efficacy in the treatment of pediatric hyperkalemia, particularly among children with CKD stages 4–5 and those on dialysis [13]. In a retrospective case series including patients aged 6 months to 18 years, SZC significantly reduced serum potassium in both acute and chronic settings without serious drug-related adverse events [13]. It also showed good gastrointestinal tolerability, stable serum sodium, no clinically relevant effect on blood pressure, and better palatability than SPS. Importantly, treatment was associated with greater dietary flexibility in some children who had previously been subjected to marked potassium restriction, although this observation comes from limited observational pediatric data [12, 13, 16, 18, 20].

In adults, SZC has been evaluated extensively in randomized trials and extension studies, with sustained efficacy in lowering and maintaining serum potassium; parallel efforts are now testing its role as a facilitator of RAASi optimization in high-risk HF and CKD populations [10, 11, 24]. By contrast, the pediatric literature remains confined largely to case series and observational reports [12], reflecting a broader lag in pediatric evidence generation. Broader pediatric literature supports the importance of hyperkalemia prevention and individualized dietary management in CKD, but it does not yet provide robust prospective SZC-specific data [2, 12, 20, 21]. This gap limits certainty and leaves children dependent on older therapies despite early signals that better-tolerated options may be available.

Given the current absence of completed large-scale pediatric clinical trials, and despite consistent adult safety and efficacy data alongside favorable initial results in children, compassionate or off-label SZC use should be framed as a cautious, individualized option rather than an established pediatric strategy [12, 13]. This may be particularly relevant in scenarios of dietary management failure, recurrent hyperkalemia, or when inadequate potassium control precludes the introduction or maintenance of reno- and cardioprotective therapies such as ACEIs or ARBs [10, 11]. Treatment should remain individualized, based on a careful balance between the toxicity risks of conventional resins and the potential benefits of SZC. Any such use should occur under close monitoring while prospective studies and the ongoing phase 3 pediatric trial further define the role of SZC in this age group [28].

In this context, SZC should be viewed as a promising but still incompletely defined pediatric option whose role requires prospective study focused on efficacy, safety, diet quality, adherence, and RAASi enablement.

## Supplementary Information

Below is the link to the electronic supplementary material.

Graphical abstract (PPTX 2.16 MB)

## Data Availability

Data sharing is not applicable to this article as no datasets were generated or analyzed during the current study.

## Abbreviations

ACEIs: Angiotensin-converting enzyme inhibitors
ARBs: Angiotensin receptor blockers
CAH: Congenital adrenal hyperplasia
CKD: Chronic kidney disease
CPS: Calcium polystyrene sulfonate
ELBW: Extremely low birth weight
HF: Heart failure
MRAs: Mineralocorticoid receptor antagonists
NOHK: Non-oliguric hyperkalemia
OR: Odds ratio
PHA1: Pseudohypoaldosteronism type 1
PK/PD: Pharmacokinetics/pharmacodynamics
RAASi: Renin–angiotensin–aldosterone system inhibitors
SPS: Sodium polystyrene sulfonate
SZC: Sodium zirconium cyclosilicate
